# Global health governance in the sustainable development goals: Is it grounded in the right to health?

**DOI:** 10.1002/gch2.1022

**Published:** 2017-01-10

**Authors:** Remco Van de Pas, Peter S. Hill, Rachel Hammonds, Gorik Ooms, Lisa Forman, Attiya Waris, Claire E. Brolan, Martin McKee, Devi Sridhar

**Affiliations:** ^1^ Department of Public Health Institute of Tropical Medicine, Antwerp Antwerpen 2000 Belgium; ^2^ Clingendael Institute The Hague Den Haag 2509 AB The Netherlands; ^3^ School of Public Health University of Queensland Brisbane 4072 Queensland Australia; ^4^ Faculty of Law, Law and Development Research Group University of Antwerp Antwerpen 2000 Belgium; ^5^ Heidelberg University Hospital Institute of Public Health Heidelberg Baden‐Württemberg 69120 Germany; ^6^ University of Toronto Dalla Lana School of Public Health Toronto Ontario M5T3M7 Canada; ^7^ University of Nairobi Nairobi Kenya; ^8^ London School of Hygiene and Tropical Medicine London UK; ^9^ University of Edinburgh Centre for Global Health Research Edinburgh EH8 9YL UK

**Keywords:** Ebola, global health, global health governance, human rights, right to health, Sustainable Development Goals

## Abstract

This paper explores the extent to which global health governance – in the context of the early implementation of the Sustainable Development Goals is grounded in the right to health. The essential components of the right to health in relation to global health are unpacked. Four essential functions of the global health system are assessed from a normative, rights‐based, analysis on how each of these governance functions should operate. These essential functions are: the production of global public goods, the management of externalities across countries, the mobilization of global solidarity, and stewardship. The paper maps the current reality of global health governance now that the post‐2015 Sustainable Development Goals are beginning to be implemented. In theory, the existing human rights legislation would enable the principles and basis for the global governance of health beyond the premise of the state. In practice, there is a governance gap between the human rights framework and practices in global health and development policies. This gap can be explained by the political determinants of health that shape the governance of these global policies. Current representations of the right to health in the Sustainable Development Goals are insufficient and superficial, because they do not explicitly link commitments or right to health discourse to binding treaty obligations for duty‐bearing nation states or entitlements by people. If global health policy is to meaningfully contribute to the realization of the right to health and to rights based global health governance then future iterations of global health policy must bridge this gap. This includes scholarship and policy debate on the structure, politics, and agency to overcome existing global health injustices.

## Impact Statement

The European Union funded Go4Health research consortium researched the positioning of health in the Sustainable Development Goals providing expert commentary, policy advice, and engagement with key global, national, and local stakeholders. Go4Health critically examined national and multilateral input into the Sustainable Development Goals process, including the proposed governance structures for health. It advocated for the inclusion of human rights in the Sustainable Development Goals through a right‐to‐health framing in its analysis and advocacy. In line with the European Commission and World Health Organization, it argued that Universal Health Coverage best embodied these values and provided rigorous academic arguments, grounded in international law, and public health to support this approach.

This research article aims to stimulate debate on the governance and implementation of global health policy frameworks such as the UHC2030 alliance and the High‐Level Political Forum on sustainable development. The possible impact of the research is to advance the normative thinking and development of transnational global health policies that safeguard and advance human rights while acknowledging that the current political momentum is limited. However, we believe that transnational, shared, health risks will eventually also lead to shared transnational health responsibilities. This manuscript, and other publications by Go4Health, will contribute to develop such cosmopolitan health governance frameworks.

## Introduction

As governments pursued the Millennium Development Goals (MDGs), the idea of global health featured increasingly in health policy literature. Academic debate has sought to define it, differentiating it from international health (Koplan et al., 2009; Fried et al., 2010), to assert its position within a public health epistemology (Brown et al., 2006), to argue for its distinctive complexity (Hill, 2011), and to contest its framing as a recent and novel phenomenon (Fidler, 2001). During this time, its use has increased exponentially, being used by public and private stakeholders, in networks and alliances, and diverse relationships, leading Kickbusch and Szabo to characterize it as a “global public health domain” (Kickbush & Szabo, 2014), with key health challenges faced by the international community being recast as issues of governance rather than disease (Kickbusch, 2006).

The management of this rich interdependence of actors, networks, and interfaces demands fresh imagining of governance. Fidler's inclusive definition of global health governance as “the use of formal and informal institutions, rules, and processes by states, intergovernmental organizations, and non‐state actors to deal with challenges to health that require cross‐border collective action to address effectively” (Fidler, 2010), has been parsed further by Kickbusch and Szabo (Kickbush & Szabo, 2014). They distinguish three global health governance concepts:

- *global health governance*, focussing on institutions and processes of global governance with an explicit health mandate such as the World Health Organization (WHO) or the Global Fund to fight AIDS, Tuberculosis, and Malaria (Global Fund);
- *global governance for health*, that embraces institutions and processes with direct or indirect impact, including the United Nations (UN), and the World Trade Organization (WTO); and
- *governance for global health*, referring to the mechanisms and institutions created at national and regional levels to support global health governance (Frenk & Moon, 2013).

But, as Frenk and Moon point out: “Global governance is distinct from national governance in one critical respect: there is no government at the global level.” (Frenk & Moon, 2013) There is a largely unchallenged acceptance of the Westphalian arrangement of populations into nation states, but as of yet no equivalent consensus around a “hierarchical political authority, or world government” with authority over them (Fidler, 2010).

If there were such a government, Owen Barder would characterize it as a failed state – as he did in a recent presentation to the London School of Economics' Diplomacy Commission:

no rule of law with no institutions to set or enforce rules, and no way to agree and enforce contracts… no mechanism to raise money for, or to deliver effectively, public goods such as clean air, law and order, financial stability, public infrastructure, research and development or disease surveillance… a winner‐takes‐all economy… with no collective insurance for its citizens against natural disasters, and in which inequality is allowed to grow to the extent where the rich have to wall themselves off from the poor (Barder, 2014).

The critique is not without substance. Yet, however imperfect, the nation‐state remains the primary locus of political legitimacy and the pursuit of justice. Indeed, as the recent referendum on the United Kingdom's EU membership, as well as the growth of anti‐European parties in France and other parts of Europe shows, there is evidence of a retreat from supranational structures.

Thomas Nagel has argued that the path from global anarchy – the absence of global authority – to global justice will not always be equitable (Nagel, 2005). It is through the expansion of complex multilateral networks and supranational arrangements between those states, initially pursuing common interests rather than altruistic sacrifice, that global governance arrangements will become institutionalized. To apply this to global health: it is likely that the global institutions that emerge may lack legitimacy, and by prioritizing the interests of its major funders (both states and non‐states) may distort distributive justice – a key critique of global health philanthropists or global public private initiatives for health (Stuckler et al., 2011). The moral and public imperative will be to democratize and hold accountable such institutions in order to enhance their legitimacy (Stuckler et al., 2011).

It is in this context that Frenk and Moon identified four essential functions of the global health system, that we will argue, parallel several key functions of the state: the production of global public goods, the management of externalities across countries, the mobilization of global solidarity, and stewardship (Fidler, 2010). With the recent UN acceptance of the Sustainable Development Goals (SDGs) (UN, ), 1United Nations Sustainable Development Goals. UN: New York 2015. https://sustainabledevelopment.un.org/topics. governance of that global health system is increasingly important. The SDGs are universal in nature, integrating economic and social development, and environmental change, with broad implications for global health. Sustainable Development Goal 3 (SDG 3) “Ensure healthy lives and promote well‐being for all at all ages” extends its claim from the unfinished agenda of the MDGs to include additional communicable disease targets, but also to address non‐communicable disease, mental health and well‐being, motor‐vehicle trauma, and the health consequences of environmental pollution. In fact, SDG 3 arguably embraces all the dominant contributors to the global burden of disease (Global Burden of Disease Study 2013 Collaborators, 2015). This near comprehensive scope, with its increasing engagement with sustainable change in other sectors, the demands SDG 3 sets for all states, and the demand for solidarity between them – makes the achievement of a system of global governance for health an imperative.

In our research, examining the positioning of health within the emerging post‐2015 SDGs, the Go4Health (*Goals and Governance for Health in the Post‐2015 Agenda*) research team has already argued that the global goal for health must be grounded in the right to health (Ooms et al., 2013). However, while the right to health may be implicit in the aspirations of Universal Health Coverage (UHC) (Ooms et al., 2014), the global health governance that will respond to the complex demands of the SDGs is yet to emerge.

In this paper, we will explore the extent to which global health governance – in the context of the early implementation of the SDGs – is grounded in the right to health. First, we will unpack the essential components of the right to health in relation to global health. We will then outline Frenk and Moon's four functions – reordered for the purposes of this analysis of global health governance, and considered in the light of the right to health – and conduct a normative analysis of how each of these governance functions should operate. We will then map the current reality of global health governance now that the post‐2015 SDGs are beginning to be implemented – a picture that may share elements of Barder's caricature – pointing to the incremental but achievable steps that are needed as we launch onto “[t]he road to dignity by 2030: ending poverty, transforming lives and protecting the planet” (United Nations Secretary‐General, ). 2United Nations Secretary‐General. The road to dignity by 2030: ending poverty, transforming lives and protecting the planet. Synthesis Report of the Secretary‐General on the post‐2015 sustainable development agenda. New York, United Nations, (2014).http://www.un.org/ga/search/view_doc.asp?symbol=A/69/700&Lang=E.

**Impact Box**

From 2012 to 2016, the EU funded Goals and Governance for Global health (Go4Health) research consortium— (http://www.go4health.eu/) — researched the positioning of health in the Sustainable Development Goals (SDGs), providing expert commentary, policy advice and engagement with key global, national and local stakeholders. Go4Health included 13 institutions, distributed between the Global North and South, including academic institutions and non‐governmental organizations (NGOs) and spanning 5 global regions. Go4Health researchers explored the health priorities of marginalized communities through multiple community consultations in different regions of the world and critically examined national and multilateral input into the SDG process, including the proposed governance structures for health. Importantly for European values, it advocated for the inclusion of human rights in the SDGs, through a right‐to‐health framing in its analysis and advocacy. In line with the European Commission and World Health Organization, it argued that Universal Health Coverage (UHC) best embodied these values and provided rigorous academic arguments, grounded in international law and public health, to support this approach. The strength of the Go4Health collaboration arose from its blend of institutional diversity (academics and NGOs) regional diversity and rich multidisciplinary dialogue— combining public health, economics, health systems and policy analysis, international relations, international law and human rights. The policy literature and debate around the SDGs has been substantially shaped by Go4Health's substantial academic output, including publications and participation at multiple conferences. In terms of metrics, Go4Health was extremely productive, with more than 50 peer reviewed articles published from its research which provide evidence for decision makers, researchers and funders to advance on their SDG promises in the coming years.

This research article is one of the final papers produced by Go4Health. Through a global health systems framework it analysis whether global health policies as part of the SDGs includes human rights considerations and provides recommendations for governance arrangements in the implementation of the SDGs. This research aims to stimulate policy debate on the governance and implementation of global health policy frameworks such as the UHC2030 alliance and the High‐Level Political Forum on sustainable development. The possible impact of the research is to advance the normative thinking and development of transnational global health policies that safeguard and advance human rights while acknowledging that the current political momentum is limited. However, we believe that transnational, shared, health risks will eventually also lead to shared transnational health responsibilities. This manuscript, and other publications by Go4Health, will contribute to develop such cosmopolitan health policy frameworks.

## Defining the Right to Health in the Context of the SDGs

By the right to health, we are referring to the entitlement of all humans to organized efforts by society that promote and improve health and the corresponding obligations born by governments and the international community, as enshrined in international human rights law (Ooms et al., 2014). While several treaties have addressed the right to health, our primary reference is the International Covenant on Economic, Social and Cultural Rights (ICESCR) (United Nations General Assembly: International Covenant on Economic, Social and Cultural Rights, 1966). This is because of its broad endorsement by states and wider scope than treaties that focus on the right to health for specific groups – like the Convention on the Rights of the Child or the Convention on the Elimination of All Forms of Discrimination against Women. The ICESCR affirms “the right of everyone to the enjoyment of the highest attainable standard of physical and mental health” (article 12(1)) and the responsibility of every state “to take steps, individually and through international assistance and co‐operation, especially economic and technical, to the maximum of its available resources, with a view to achieving progressively the full realization of” the right to health (article 2(1)) (United Nations Committee on Economic, Social and Cultural Rights, 2003). These articles have been interpreted to codify rights to both adequate health care and the underlying determinants of health, and to place corresponding obligations on governments to act on health at home and where able, abroad (United Nations Committee on Economic, Social and Cultural Rights, 2000).

A right to health framed global governance for health would need to ensure at least “minimum essential levels of each of the rights enunciated in the Covenant, including essential primary health care” – rights expressed in the proposed SDGs as the achievement of UHC “including financial risk protection, access to quality essential health care services, and access to safe, effective, quality and affordable essential medicines, and vaccines for all”.^1^ However, the right to health is not the only economic, social, or cultural right impacting on the SDGs' implementation, which also require realization of rights to water and sanitation, food, housing, education, and collectively, to development. This broader scope is exemplified by the content of the inter‐related goals that are necessary for the implementation of the health goals: for instance, SDG 2 (End hunger, achieve food security, and improved nutrition and promote sustainable agriculture); SDG 4 (Ensure inclusive and equitable quality education and promote lifelong learning opportunities for all); SDG 6 (Ensure available and sustainable management of water and sanitation for all); SDG 11 (Make cities and human settlements inclusive, safe, resilient and sustainable); SDG 16 (Promote peaceful and inclusive societies for sustainable development, provide access to justice for all and build effective, accountable and inclusive institutions at all level); and SDG 17 (Strengthen the means of implementation and revitalize the global partnership for sustainable development).

## Four Functions for Global Health Governance in the Context of the SDGs: A Normative Right to Health Analysis

With their claim to universality, the SDGs do provide a framework within which global health is redefined in terms of the health of the global population, understood in terms of global interdependence. The absence of a world government does not obviate the need for global governance, although the form of that governance will clearly be different to the governance of nation states. To facilitate our analysis, we have re‐ordered and adapted Frenk and Moon's four functions of the global health system (Frenk & Moon, 2013), building on the current functions of nation states that would allow the right to health to be achieved, and extrapolating them to global governance for health:

1. *Stewardship* provides “overall strategic direction to the global health system” (Frenk & Moon, 2013), and embodies in many ways the functions of the executive branch of the state: the establishment of norms, values, and rules that guide the development of policy and setting of priorities, the advocacy for global health across sectors and the convening of partnerships at global and regional level that might enable its achievement.
2. *The production of global public goods* is instrumental in progressively ensuring “the highest attainable standard of physical and mental health” (Frenk & Moon, 2013), and embodies and operationalises the policy concepts elucidated in the stewardship function. Arguably this parallels the functions of the legislative branch of the state, implementing policy with the resources mobilized domestically and through global solidarity. Frenk and Moon draw particular attention to knowledge‐related public goods, research and development, standards and guidelines, comparative evidence, and analyses. We define global public goods more broadly, echoing Kickbusch's call for an expansive concept of global public goods for health (GPGH) which highlights health's “deep relation to human rights, equity and governance” noting they “all relate to the provision of GPGH” (Kickbusch, 2013).
3. *The mobilization of global solidarity* combines four major sub‐functions: the shared financing of global health; capacity building and technical assistance; humanitarian interventions in crisis; and agency for the marginalized and dispossessed. This function parallels the role of the state in revenue raising through taxation and other means, coupled with resources provided by global partners, and its disbursement in the implementation of redistributive policies determined through its stewardship functions.
4. *The management of externalities* embraces those functions that contain the negative impact of decisions made by one state – or transnational body – on others. Frenk and Moon list the deployment of instruments such as surveillance systems, coordination mechanisms, and information channels essential for controlling international risk, but the exercise of sanctions – analogous to the judicial branch of the state, would need to find equivalence at the global level.

In terms of *stewardship*, a Right to Health driven Global Health Governance would align its goal with Article 12(1) ICESCR which recognizes “the right of everyone to the enjoyment of the highest attainable standard of physical and mental health” (United Nations General Assembly: International Covenant on Economic, Social and Cultural Rights, 1966). It would achieve this by taking incremental steps “to the maximum of its available resources, with a view to achieving progressively the full realization of the rights recognized in the present Covenant…” (United Nations General Assembly: International Covenant on Economic, Social and Cultural Rights, 1966). But it would define “minimum core obligations” from which this progressive realization would proceed, ensuring “the right of access to health facilities, goods and services on a non‐discriminatory basis, especially for vulnerable or marginalized groups… access to the minimum essential food which is nutritionally adequate and safe, to ensure freedom from hunger to everyone… access to basic shelter, housing and sanitation, and an adequate supply of safe and potable water… [t]o provide essential drugs… [t]o ensure equitable distribution of all health facilities, goods and services… [t]o adopt and implement a national public health strategy and plan of action, on the basis of epidemiological evidence, addressing the health concerns of the whole population” (United Nations Committee on Economic, Social and Cultural Rights, ). 3United Nations Committee on Economic, Social and Cultural Rights. General Comment No. 14: The Right to the Highest Attainable Standard of Health, E/C.12/2000/4. Geneva: United Nation. (2000). http://www.refworld.org/docid/4538838d0.html. This non‐discriminatory health strategy – under global governance – would be “devised, and periodically reviewed, on the basis of a participatory and transparent process” (United Nations Committee on Economic, Social and Cultural Rights, 2000).^3^

Under a Right to Health based system of global health governance, the *production of global public goods* would necessarily prioritize meeting those “minimum core obligations”, delivering the knowledge‐related public goods that ensure universal access to effective curative and preventive health services and the essential public health provisions anticipated in the ICESCR (United Nations Committee on Economic, Social and Cultural Rights, 2000).^3^

The *mobilization of global solidarity* would be integral to the achievement of this. Under the Right to Health, every state is responsible for ensuring these minimum core obligations “to the maximum of its available resources” (United Nations General Assembly: International Covenant on Economic, Social and Cultural Rights, 1966). 4United Nations General Assembly: International Covenant on Economic, Social and Cultural Rights, G.A. Res. 2200A (XXI), 21 U.N. GAOR Supp. (No. 16) at 49, U.N. Doc.A/6316 (1966), 993 U.N.T.S. 3, entered into force. 1976. http://www1.umn.edu/humanrts/instree/b2esc.htm. It assumes shared financing of global health as states “take steps, individually and through international assistance and co‐operation, especially economic and technical, to the maximum of its available resources” (United Nations General Assembly: International Covenant on Economic, Social and Cultural Rights, 1966),^4^ to achieve progressively the realization of the Right to Health. Shared responsibility to realize the right to health is emphasized in General Comment 14 which suggests that “it is particularly incumbent on States parties and other actors in a position to assist, to provide ‘international assistance and cooperation, especially economic and technical’ which enable developing countries to fulfil their core and [comparable priority obligations]”. 5United Nations Committee on Economic, Social and Cultural Rights. General Comment 14: The right to the highest attainable standard of health. (Twenty‐second session, 2000), U.N. Doc. E/C.12/2000/4 (2000), reprinted in Compilation of General Comments and General Recommendations Adopted by Human Rights Treaty Bodies, U.N. Doc. HRI/GEN/1/Rev.6 at 85 (2003). http://www1.umn.edu/humanrts/gencomm/escgencom14.htm. Subsequent expert interpretations have emphasized that states hold extraterritorial obligations to enable the realization of “core obligations to realize minimum essential levels of economic, social and cultural rights” (International Commission of Jurists, 2012; Hammonds et al., 2012). In capacity building and technical assistance, and in particular in humanitarian interventions in crisis, this shared responsibility is assumed. The “agency for the marginalized and dispossessed”, to which Frenk and Moon point is subsumed in the right to health principle of non‐discrimination, and in its prioritization of vulnerable and marginalized groups. Here the bar is raised – if a health issue disproportionately affects the marginalized, protection of their interests necessitates a policy response, even if, at a population level, it is not cost‐effective (or politically palatable at the domestic level) (Ooms et al., 2014).

The *management of externalities* under a right to health based global health governance would be implied in the principle of shared responsibility, and interface with the recognition of other cognate rights articulated in the Universal Declaration of Human Rights, 6United Nations General Assembly: Universal Declaration of Human Rights, G.A. res. 217A (III), U.N. Doc A/810 at 71. 1948. Available from: http://www1.umn.edu/humanrts/instree/b1udhr.htm. and in the ICESCR (United Nations General Assembly: International Covenant on Economic, Social and Cultural Rights, 1966). This could imply that the legal functions of the WHO, specifically the legislative and executive authority, in addressing global health threats should be strengthened. One can think here of deepening the International Health Regulations (IHR) (Gostin & Sridhar, 2014), or even a much further reaching Framework Convention on Global health, that would serve as a legal umbrella for the further management responsibilities of states to address global “bads” and strengthening GPGH (Gostin et al., 2013).

## Global Health Governance in the Context of the SDGs and the Right to Health

### Stewardship

In terms of *stewardship* for global health within the context of the SDGs – setting the global health agenda, establishing norms and guidelines, engaging partners for international policy development and implementation – WHO is unique in terms of its legitimacy as the only global health institution with a mandate to promulgate international law (Moon et al., 2010). Health goal SDG 3 “Ensure healthy lives and promote well‐being for all at all ages” echoes both the right of everyone to “highest attainable standard of physical and mental health”,^4^ and WHO's definition of health as a “state of complete physical, mental and social well‐being and not merely the absence of disease or infirmity”. 7Preamble to the Constitution of the World Health Organization as adopted by the International Health Conference, New York, 19–22 June, 1946; signed on 22 July 1946 by the representatives of 61 States (Official Records of the World Health Organization, no. 2, p. 100) and entered into force on 7 April 1948.www.who.int/governance/eb/who_constitution_en.pdf. There is clear synchrony in aspiration. But despite the representation of nation states through their ministers of health in the World Health Assembly (WHA), and respect for its norm setting functions, the capacity of the WHO to embody the stewardship function of global governance for health is repeatedly questioned (Hoffman & Røttingen, 2014; Ruger & Yach, 2009). Substantially under‐resourced, and operationally hamstrung, WHO faces a situation where the bulk of its budget is earmarked by powerful “donor” states. Sridhar and Woods have phrased this institutional gridlock as “trojan multilateralism”, defined as “*increased funding to multilateral institutions that is creating the illusion of multilateral intent*, *whereas it is covertly introducing bilateral goals and interests into multilateral institutions*” (Sridhar & Woods, 2013).

As a consequence, WHO is constrained in terms of policy and direction, and there are equivocal perceptions of its capacity to drive the global health agenda. This was most recently evident in the critiques of its executive role in and leadership response to the Ebola outbreak (Gostin et al., 2014), and again in its failure to secure UHC as the overall SDG health goal (Brolan & Hill, 2015). At the same time, recognition of the centrality of WHO to global health governance is evident in proposals for a Committee C which would allow WHO to more effectively engage civil society, formalizing civil society's current significant contribution to global health governance (Kickbush et al., 2010). Yet recent proposals for a new UN agency to address global health (Dybul et al., 2012), revisit earlier proposals to extend the Global Fund from its targeted communicable disease mandate to become a Global Fund for Health (Cometto et al., 2009), and an earlier UN decision that relocated management of the HIV epidemic from WHO into the Joint United Nations Programme on HIV/AIDS (UNAIDS). 8UNAIDS. (2016) http://www.unaids.org/.


But WHO has not held a monopoly on the stewardship for global health for some time: the UN agencies UN Children's Fund (UNICEF), UN Population Fund (UNFPA), and UNAIDS have specific global health mandates that interface with WHO. Since the 1990s, the World Bank has also made the claim for investing in health. 9World Bank. Pandemic Emergency Financing Facility: Frequently Asked Questions (2016) http://www.worldbank.org/en/topic/pandemics/brief/pandemic-emergency-facility-frequently-asked-questions. The WTO exercises a governance role for medicines through the Agreement on Trade‐Related Aspects of Intellectual Property Rights (TRIPS). 10World Trade Organization (WTO) ‐ Agreement on Trade‐Related Aspects of Intellectual Property Rights (TRIPS Agreement).(1994) http://www.wipo.int/wipolex/en/other_treaties/details.jsp?treaty_id=231. The Organization for Economic Cooperation and Development (OECD) – initially the provenance of high income economies – has now redefined aid effectiveness (including for health) into development effectiveness, reaching beyond its immediate membership and embracing the multiple, complex contributors to global health and development (Busan Partnership Agreement, 2011).

What is increasingly clear is that there will be no return to an imagining of a global governance hierarchy and that the concrete, architectural metaphors of the past no longer suffice (Fidler, 2009). Global health governance will continue to be networked, with largely voluntary partnerships and alliances addressing key issues as they have in the GAVI Alliance, Global Fund, Roll Back Malaria, The Partnership for Maternal, Newborn & Child Health, and the NCD‐Alliance. Under the right to health, coordination of the networks would itself be a necessary function, the global policies regularly and transparently re‐evaluated (United Nations Committee on Economic, Social and Cultural Rights, 2003). This option has been canvassed in the form of a Global Health Forum, offering voice to multiple stakeholders, beyond the state representation of the UN system. 11WHO World Health Forum. Concept paper. (2011) http://www.who.int/dg/reform/en_who_reform_world_health_forum.pdf. The conspicuous consultation of the “World We Want” campaign 12United Nations. World We Want. (2016). https://www.worldwewant2030.org/. was a direct response to civil society's absence from the formulation of the MDGs.

The experience of the MDGs is that, once accepted, the goals and their targets are relatively fixed. Despite their significant contribution to the Global Burden of Disease (Murray et al., 2013), the non‐communicable diseases (NCDs) were marginalized for the 15 years of the MDGs. Although NCDs are now included in the SDGs, it is to be seen whether they will receive the prominence and attention deserved. The NCD challenge does not only require funding or new financing mechanism, but also global regulation to address the key vectors of the epidemic, such as the overconsumption of sugars, tobacco, and alcohol. The global governance structures as part of the sustainable development agenda are poorly suited to deal with this multisectoral issue (Sridhar et al., 2013).

The MDG5b “Achieve, by 2015, universal access to reproductive health” was only added in 2007 following persistent community protest. The SDGs may provide for the progressive realization envisaged in the right to health, but experience from the MDGs suggests the SDG indicators currently under development will determine the priorities for implementation, and as with the MDGs, will form the hubs around which governance structures will coalesce. In terms of ensuring support for the minimum core obligations and prioritizing the marginalized and vulnerable, this comes with some risks.

The recent Ebola crisis provides some insight into potential processes: in its aftermath, WHO's Report of the Interim Ebola Assessment Panel recommended support for national and international capacity to implement its IHR – but recognizing the reluctance of member states to raise their contributions, 13TWN Info Service on Health Issues. Health: WHO D‐G warns of serious funding shortfalls in 2016–17 budget. Published in SUNS #8346 dated 2 November (2016) Third World Network. http://www.twn.my/title2/health.info/2016/hi161102.htm. recommended a modest WHO Emergency Contingency Fund and a process of internal reform. 14WHO. Report of the Interim Ebola Assessment Panel. (2015) WHO: Geneva http://www.who.int/csr/resources/publications/ebola/report-by-panel.pdf. Concurrently, the World Bank Pandemic Emergency Financing Facility has been proposed (World Bank, 2016). The facility will provide financial resources to deploy trained health workers, equipment, medicines, and whatever else is required quickly when a pandemic hits. Simultaneously, the Global Health Security Agenda, driven by the Centers for Communicable Disease Control and Prevention and related United States' agencies, has been created in partnership with other nations, international organizations and public and private stakeholders, to “seek to accelerate progress toward a world safe and secure from infectious disease threats and to promote global health security as an international security priority.” 15US Government.The Global Health Security Agenda. (2015) http://www.globalhealth.gov/global-health-topics/global-health-security/ghsagenda.html. Each of these initiatives is a rational response to a significant issue for global health security. Each would have to recognize the legitimacy of other contributions. Yet the lack of a single locus for governance responding to this threat is of concern, and risks the duplication of effort and the disruptive lack of coordination that has characterized other acute crises. And while Ebola is a tragic threat for Sub‐Saharan Africa, the significant investment for its control from key development donors cannot be considered proportional compared with other global health burdens. But even within the response to Ebola, while accelerated investment in vaccines development has been highlighted, the health systems deficits identified as underlying the outbreak may not obtain the urgent financial and technical attention required. A short‐term focus on a vaccine may deflect commitment from the long term support necessary to address the lack of development and coherence between elements of the systems building blocks – the health workforce, health financing mechanisms, governance and stewardship, and health information systems (Gostin & Friedman, 2015). The policy and governance responses addressing global health security will be amongst many arenas where the competing interests in networked governance may challenge that essential stewardship function that would protect right to health values.

The right to health concept of non‐discrimination also appears to differ from the commitment to address inequality foreshadowed by the SDGs. From the report of the High Level Panel, 16Report of the UN Secretary‐General's High‐level Panel of Eminent Persons on the Post‐2015 Development Agenda. (2013) http://www.un.org/sg/management/pdf/HLP_P2015_Report.pdf. the dictum “leave no one behind” has been one of the “transformative elements” of the SDGs, articulated in SDG 10 “Reduce inequality within and among countries”.^1^ It is also included in other goals such as SDG 5 “Achieve gender equality and empower all women and girls”.^1^ But marginalized and vulnerable populations have not been explicitly identified within the SDGs and Vandermoortele points to the consequences of the SDGs' primary focus on poverty as the underlying concept of global equity (Vandemoortele, 2015). It is in this concept of equity, however, that the right to health, because of its state‐centric orientation, produces unexpected outcomes when applied to global governance. As the analysis of UHC and the Right to Health pointed out (Ooms et al., 2014), while the Right to Health expects rectification of inequalities within states, and the privileging of marginalized groups – arguably including refugees and asylum seekers(Brolan et al., 2015) – it does not apply that expectation *between* states. The principle of shared responsibility in the Right to Health requires the international solidarity that would ensure a low‐income country meets the minimum standard for provision of health services, but at a global level, it does not compellingly articulate expectations of equity beyond that.

### The production of global public goods

The production of global public goods, as we define it in a broader sense, operationalizes the abovementioned stewardship functions, and is instrumental in progressively realizing the Right to Health's “highest attainable standard of physical and mental health” – SDG 3's healthy lives and well‐being for all. The norms and guidelines for global health are detailed in SDG 3 targets: achieving UHC, through health systems that are adequate resourced and staffed, and guarantees protection against financial risk, access to quality essential health care services, sexual and reproductive health care, and essential medicines for all.^1^ But what the SDGs and their targets do not do is to articulate a clear set of minimum core obligations. For some targets, absolute levels are asked of each state: in SDG 3.2 all countries are to aim “to reduce neonatal mortality to at least as low as 12 per 1000 live births and under‐5 mortality to at least as low as 25 per 1000 live births”; SDG 3.7 seeks “universal access to sexual and reproductive health‐care services, including for family planning, information and education”. Other targets are expressed at the global level: SDG 3.1 is specific in aiming for “a global maternal mortality ratio to less than 70 per 100,000 live births”, with national targets yet to be established; SDG 3.6 seeks to halve global road traffic deaths within 5 years. But other goals lack sufficient operational definition.^1^ SDG 3.3 unrealistically proposes ending epidemics of communicable diseases; SDG 3.4 the promotion mental health and well‐being, but without specifying a level of achievement; SDG 3.5 broadly advocates strengthening the prevention and treatment of substance and alcohol abuse; SDG 3.9 seeks to substantially reduce morbidity and mortality from pollution – again without quantification. And while the targets have expanded on the narrower MDG focus, they are not comprehensive. For example, it has been argued that the use of “premature” mortality diminishes the attention given to older people (Lloyd‐Sherlock et al., 2016). A defined set of minimum core obligations, required to satisfy the Right to Health, is not set by the SDGs, although it may be implied in SDG 3.8 “Achieve UHC”. By extension, the tracking of progressive realization of those elements that are ultimately operationalised in the SDGs will be limited to those that benefited from indicators agreed by the Inter‐agency and Expert Group on Sustainable Development Goal Indicators (IAEG‐SDGs).

Sridhar and colleagues have elaborated on the indicators required for UHC monitoring in the SDGs that would cover the six legal principles of the right to health (Sridhar et al., 2015). These six principles include progressive realization of the Right to Health and fulfilment of the minimum core obligations, cost‐effectiveness, non‐discrimination, shared responsibility, participatory decision making and attention to vulnerable, and marginalized groups. Ooms and colleagues have assessed the UHC framework as being in line with the legal principles of progressive realization. The non‐discrimination principle is addressed via the development of a health system that is accessible to all, including financially accessible at the point of service. The cost‐effectiveness principle might be addressed if UHC follows national determined sets of health services. However, participatory decision making and prioritizing marginalized and vulnerable groups is only included to a limited extent in the UHC framework and its indicators. The biggest difference is that the Right to Health principles of minimum core obligations and shared responsibility, in the form of international financial assistance, receive no attention in UHC policies (Ooms et al., 2014).

The IAEG‐SDGs has developed an indicator framework for the monitoring of the goals and targets of the post‐2015 development agenda at the global level, and to support its implementation. 17UN DESA's Statistics division. Second meeting of the Inter‐agency and Expert Group on Sustainable Development Goal Indicators. (2016) http://unstats.un.org/sdgs/meetings/iaeg-sdgs-meeting-02. The World Bank and WHO consider it critical to have two indicators on the UHC target 3.8; one of the coverage of interventions, and one on financial protection, both with an explicit equity dimension. 18UN DESA's Statistics division. Second meeting of the Inter‐agency and Expert Group on Sustainable Development Goal Indicators. Statement on SDG 3 Joint Statement by WHO and UNICEF on behalf of health agencies. (2016) http://unstats.un.org/sdgs/files/meetings/iaeg-sdgs-meeting-02/Statements/UNSSO%20statement_Goal%203%20-%20Oct%202015.pdf. The World Bank and WHO have released a first global UHC monitoring report in 2015 that is built around these two main indicators. The coverage indicator looks both to prevention services and treatment while their proposed financial protection indicator was built around two sub‐indicators: the incidence of impoverishment resulting from OOP health payments, and the incidence of financial catastrophe from the same cause. 19World Health Organization & World Bank. Tracking universal health coverage: first global monitoring report. (2016) .http://apps.who.int/iris/bitstream/10665/174536/1/9789241564977_eng.pdf?ua=1. But to the dismay of civil society organizations, and also the WHO and the World Bank, the IAEG‐SDGs has suggested to change the SDG 3.8 financial protection indicator in “number of people covered by health insurance or public health system per 1000 population”, an indicator that is not a valid measure of financial risk protection and could hide existing health inequalities in countries (Revelo, 2016).

The research and development of vaccines and medicines, an essential global public good for securing essential medicines for all, is the explicit focus of SDG 3b, and recurs in SDG 3.8 “Achieve UHC”. The explicit inclusion of the TRIPS agreements and the flexibilities to protect Low and Middle Income Countries, speaks to an increasingly contested arena for pharmaceutical research, production and access, in debates on trade partnerships such as the Trans Pacific Partnership (TPP), 20Trans‐Pacific Partnership. International trade association. (2016) http://www.trade.gov/fta/tpp/. and renewed calls for tiered pricing of drugs (Williams et al., 2015). Public health scholars have argued for a Global Biomedical Research & Development (R&D) Fund that would address Anti‐microbial Resistance, emerging infectious diseases, and neglected diseases, incorporating financing and coordination mechanisms D that deliver both innovation and access to medicines and technology by the poor (Balasegaram et al., 2015).

Implicit in these governance and policy proposals are issues of cost and cost‐effectiveness, and implications for availability, accessibility, acceptability, and quality. While the preamble to the SDGs envisages “a world of universal respect for human rights and human dignity, the rule of law, justice, equality and non‐discrimination”.^2^ the Right to Health principle of non‐discrimination, while applying within state jurisdictions, has not been extrapolated to apply between them.

Another public good required on the path to UHC is the health workforce: SDG 3c mentions to substantially increase health financing and the recruitment, development, training and retention of the health workforce in developing countries, especially in least‐developed countries and small island developing states. Despite the recognition since the early 2000s that the health workforce is a crucial bottleneck in attaining the health‐related MDGs – and the concerted commitment to building that workforce – the global health workforce gap has grown, with a current estimated global deficit of 7.2 million health workers. Because of demographic and epidemiological changes, this deficit is expected to grow to 12.9 million health workers by 2035 (Sidibé & Campbell, 2015), further accentuated by maldistribution and urban bias. The same governance debate developed in relation to access to essential medicines, 21The United Nations Secretary‐General's High‐Level Panel on Access to Medicines (2016). Available at: http://www.unsgaccessmeds.org/#homepage-1. needs to be happen for equitable distribution and just policies for health workforce development.

Global public goods in terms of knowledge generation have been acknowledged in the call for a data revolution to underpin the monitoring and reporting functions for the SDG indicators. 22UN Data revolution group (2016) Available at http://www.undatarevolution.org/. SDG 16, promoting peaceful and inclusive societies for sustainable development, includes the provision of legal identity for all, dependent on comprehensive vital registration systems. The Global Burden of Disease report has been useful in quantifying health priorities, and will continue to play a role in monitoring global change (Rudan & Chan, 2015). The IN‐DEPTH network will provide an evolving platform for monitoring SDG indicators (INDEPTH Network, 2016). The systematized evaluation of other health systems evidence through meta‐analyses such as the Cochrane collaboration and the Health Observatories' Health in Transition reports are a necessary complement for understanding change (European observatory on Health Systems and Policies, 2016).

### The mobilization of global solidarity

The mobilization of global solidarity combines four major sub‐functions: development financing; technical cooperation and capacity building; humanitarian interventions in crisis; and advocacy – and agency – for the marginalized and dispossessed. While the other three sub‐functions are likewise important, a major focus for activities seeking to mobilize global solidarity should be the creation of a just form of financial redistribution between richer and poorer societies.

In the context of the SDGs, the recent Third International Conference on Financing for Development (FfD3) offers some insight into proposed financing of the SDGs as a whole, 23UN 69/313. Addis Ababa Action Agenda of the Third International Conference on Financing for Development (Addis Ababa Action Agenda).(2015) http://www.un.org/ga/search/view_doc.asp?symbol=A/RES/69/313. Flavia Bustreo. Financing the health Sustainable Development Goal. (2015): http://www.who.int/life-course/news/commentaries/financing-health-sustainable-goal/en/. although the estimated SDG envelope is well beyond current projections (Bustreo, 2015). The dominant focus is on increasing domestic resourcing, “through modernized progressive tax systems, improved tax policy and more efficient tax collection” (Global Policy Watch, 2015). Illicit financial flows and corruption are targeted; international tax cooperation to be “scaled up” with emphasis being placed on public‐private partnerships but stopping short of a global institution to govern international tax issues and their fair share across the globe. The roles of the private sector – effectively directed towards an alignment with sustainable development – and the contribution of migration and empowerment of women are noted. In its state‐centric orientation it is taking the first baby step towards being consistent with the right to health's obligation for the state to “take steps, individually and through international assistance and co‐operation, especially economic and technical, to the maximum of its available resources” (United Nations General Assembly: International Covenant on Economic, Social and Cultural Rights, 1966).

But is the shared responsibility sufficiently addressed, or are we witnessing in this expanded development agenda a reluctance to sustain – let alone extend – current development assistance? The FfD3 report reiterates the need for providers of Official Development Assistance (ODA) to re‐commit to their target of 0.7% of Gross National Income – more honored in the breach than the observance – and welcomes the additional resources offered by South–South cooperation, and philanthropy. With regards to global health, the contribution of multi‐stakeholder partnerships such as GAVI, Global Fund and the Global Financing Facility in support of Every Woman, Every Child are specifically mentioned, together with WHO's role in directing and coordinating, and its contribution to health systems strengthening and the Framework Convention on Tobacco Control.

But consistent with the analysis of the fourth Organization for Economic Cooperation and Development High Level Forum on Aid Effectiveness in Busan (Fidler, 2009), aid is seen as only one contributor to development, with trade and the engagement of the private sector an increasingly dominant counterpart. The lengthy treatment of the WTO in the FfD3 – and for health, the reaffirmation of the right to TRIPS flexibilities for low income countries – suggests some anxiety around the complexity of “global solidarity” that uncritically embraces the private sector. The global partnership and solidarity of the FfD3 does not live up to the common but differentiated responsibility demanded by the right to health, and the FfD3, while identifying the diversity of potential contributors to development, not only did not offer a governance mechanism for ensuring they deliver, it specifically rejected it during the negotiations (Global Policy Watch, 2015).

### The management of externalities

Frenk and Moon identify certain functions that contain the negative impact of decisions made by one state – or transnational body – on others. They argue for deployment of instruments such as surveillance systems, coordination mechanisms and information channels to respond to international risks to health. Examples include the global alert system for infectious disease, tsunami warning systems, and monitoring of radioactivity in the atmosphere to detect nuclear power plant accidents. The Lancet–University of Oslo commission on global governance for health came to the conclusion that there are systemic global governance dysfunctions, undermining the management of externalities that impact health. The commission has identified democratic deficits, weak accountability mechanisms and poor transparency, institutional inertia, missing institutions, and an inadequate policy space for health, as key reasons why it is so difficult to manage externalities, or so the called “global bads” for health at an international level (Ottersen et al., 2014).

In most cases where there is a severe threat to health arising from direct transnational developments, such as epidemic disease, there will be consensus among the states concerned about the action to be taken. However, this will not always be the case. Examples include hesitancy in notifying outbreaks of infectious disease because of concerns about the impact on trade or tourism, with the former a factor in the delay in recognizing the West African Ebola outbreak, cross‐border movement of refugees fleeing conflict, as in Syria, or activities that restrict or contaminate cross‐border water supplies.

Were these issues arise within a state or at least one with functioning institutions, measures would be taken to enforce policies to address the fundamental problems. The scope to do so at international level is constrained by the doctrine of state sovereignty. The revised IHR permit the WHO to draw on evidence from sources other than national governments when a disease outbreak is suspected.

However, beyond the changes to the IHR, developments in global cooperation have either been of little or no help in advancing the right to health or have actually undermined it. With many armed conflicts involving countries linked to, or protected by, a permanent member of the Security Council, action is frequently vetoed (Hale et al., 2013). International trade agreements place little, if any, weight on health considerations, tending to favor the powerful, which includes many corporations producing health damaging products. Incorporation of health considerations is often cosmetic, such as the restriction on tobacco companies taking certain actions against states included in the Trans Pacific Partnership, while leaving open the possibility of associations of tobacco producers, in effect front organizations for the tobacco companies, to do so. 24Tobacco Tactics. Front Groups.(2016) http://www.tobaccotactics.org/index.php/Front_Groups.


Thus, of the four functions, the institutional arrangements necessary to achieve the right to health seem weakest here.

## Does Global Health Governance in the SDGs Satisfy the Right to Health?

The advent of economic globalization in particular has meant that some states and other global actors exert considerable influence on the realization of economic, social and cultural rights across the world. The Maastricht Principles on the Extraterritorial Obligations of States in the Area of Economic, Social and Cultural Rights clarify the legal principles for states to respect, protect, and fulfil human rights both within their domestic territories and outside their national borders (Hammonds et al., 2012). In theory, the existing human rights legislation would enable the principles and basis for the global governance of health beyond the premise of the state. In practice, there is a governance gap between the human rights framework and practices in global health and development policies. This gap can be explained by the political determinants of health that shape the governance of these global policies (Tobacco Tactics, 2016).

The central question for this paper was: does the SDG agenda overcome that gap? Does the SDG agenda entail new or improved global health governance that satisfies the demands of the Right to Health? The answer is, unfortunately, negative. In each of the four functions of global health governance (according to Frenk and Moon), the SDG health agenda undercuts the Right to Health. Firstly; the stewardship function of global health governance is not addressed in the SDGs. Secondly; the GPGH that are included in the SDGs are insufficient. Beyond domestic legislation, there is no clear allocation of the responsibility to produce those global public goods. Thirdly; the mobilization of global solidarity merely includes the long‐existing promise of High‐Income Countries to spend 0.7% of their Gross National Income on Official Development Assistance complemented by a shifting focus on trade investments and domestic financing. Lastly; the management of externalities that impact on health is hardly considered in the SDGs. All in all, the SDG agenda does not alter let alone improve global health governance.

This assessment of a relative neglect of human rights in the SDG health target is also consistent with a report on the World Bank, a major institution promoting the UHC target, by the UN Special rapporteur on extreme poverty and human rights. This rapport concludes that “the existing approach taken by the World Bank to human rights is incoherent, counterproductive and unsustainable. For most purposes the World Bank, is a human rights – free zone”. 25Report of the Special Rapporteur on extreme poverty and human rights. UNGAA/70/27. Augustus (2015) http://www.un.org/en/ga/search/view_doc.asp?symbol=A/70/274#sthash.oPzhm92I.dpuf. The implementation of the SDGs will depend on the eventual realization of the financing framework agreed at the FfD3. It attributes a significant role for the private sector in development, without providing any mechanisms by which corporations can be held accountable (Kvangraven, 2015).

On the other hand, it has been argued that the SDGs do not depart from the discourse of accountability through enumeration established in the MDGs, but rather intensify it. The number of targets has increased from 21 to 169 and the indicators are likely to proliferate accordingly. Even richer countries would struggle with the data collection. The SDGs could have an epistemic, communicative and coordinating role but to truly play a constructive role in global development it might be wise to focus on the 17 higher‐level goals, rather than the 169 targets. It might open up innovation, flexibility, and fuller democratic accountability (Ooms et al., 2014). This resonates with Kickbush's call for “a concept of global public health in the SDGs context which is democratic and ecological rather than utilitarian” (Kickbush, 2016).

Finally, legal scholars have suggested that current representations of the right to health in the SDGs are insufficient and superficial, because they do not explicitly link SDG commitments or right to health discourse to binding treaty obligations for duty‐bearing nation states or entitlements by people, whether legal citizens or undocumented migrants (Williams & Blaiklock, 2016). If global health policy is to meaningfully contribute to the realization of the right to health and to rights based global health governance then future iterations of global health policy must bridge this gap. This includes scholarship and policy debate on the structure, politics and agency to overcome existing global health injustices (Benatar, 2016).

## Authors' Contributions

PH, RP, and GO conceived the study and analytical framework. PH designed a first draft manuscript. All authors provided comments and additional content contributions. RP integrated and edited all inputs in a final manuscript. All authors reviewed and approved the final manuscript.

## Conflict of Interests

The authors declare they have no competing interests.

## Ethical Issues

Not applicable.
